# An *in silico* evaluation of lorlatinib as a potential therapy for novel amino acid substitutions in the tyrosine kinase domain of the ALK protein associated with cancer

**DOI:** 10.3389/fphar.2025.1605314

**Published:** 2025-06-18

**Authors:** Richard Junior Zapata Dongo, Julio A. Poterico, Diletta Fontana, Luca Mologni, Carla Alvarez-Chacon, Juan Rojas-Armas, Jaeson Calla

**Affiliations:** ^1^ Doctoral Programme in Health Sciences, Faculty of Medicine, Universidad Nacional Mayor de San Marcos, Lima, Peru; ^2^ Faculty of Health Sciences, Universidad de Huánuco, Huánuco, Peru; ^3^ School of Medicine and Surgery, University of Milano-Bicocca, Monza, Italy; ^4^ Faculty of Biological science, Universidad Nacional Pedro Ruiz Gallo, Lambayeque, Peru

**Keywords:** ALK, binding energy, deleterious, cancer, lorlatinib, molecular docking, SIFT, PolyPhen-2

## Abstract

The *anaplastic lymphoma kinase* (*alk*) gene on chromosome 2 encodes a receptor tyrosine kinase protein essential for key signaling pathways regulating cell proliferation and differentiation. Mutations in *alk* have been implicated in multiple cancers, including non-small cell lung cancer (NSCLC) and anaplastic large cell lymphoma. While ALK inhibitors have demonstrated efficacy in targeted therapies, resistance due to specific amino acid substitutions requires the development of novel therapeutic strategies. This study aims to identify ALK tyrosine kinase domain mutations using data from the Cancer Genome Atlas and to evaluate the potential of lorlatinib, a third-generation ALK inhibitor, in overcoming these mutations. Using the SIFT and Polyphen-2 algorithms, we identified 53 deleterious ALK mutations associated with different newly recognized cancer types. These mutations were subjected to *in silico* molecular docking with lorlatinib. Our results indicate strong binding affinities (ranging from −9.4 to −10.8 kcal/mol) across all identified mutations, suggesting a significant interaction between lorlatinib and mutated ALK variants. Furthermore, protein-ligand interaction analysis revealed critical hydrophobic interactions, hydrogen bonds, and essential halogen bonds reinforcing lorlatinib as a potential utility in treating a broader spectrum of ALK-positive tumors beyond NSCLC. This research underscores the importance of repurposing *in silico* drugs and highlights the need for continued exploration of ALK mutations in cancer therapeutics.

## Introduction

The anaplastic lymphoma kinase (*alk*) gene, located on the short arm of chromosome 2 (2p23), encodes a receptor tyrosine kinase that plays a critical role in regulating signaling pathways involved in cell proliferation, survival, and differentiation, particularly during nervous system development (Huret and Dessen, 2024; Yao et al., 2013).

The ALK fusion gene was first identified in anaplastic large-cell lymphoma (ALCL), and has since been implicated in several types of cancer, including colorectal carcinoma (CRC), inflammatory myofibroblastic tumor (IMT); B-cell lymphoma (BCL); non-small-cell lung cancer (NSCLC); non-Hodgkin’s lymphoma (NHL); neuroblastoma, and other less common malignancies (Holla et al., 2017).

Targeted therapies using tyrosine kinase inhibitors (TKIs) have shown efficacy against ALK-positive cancers, with crizotinib being the first TKI approved by the U.S. Food and Drug Administration (FDA) for NSCLC. However, resistance to crizotinib has emerged due to point mutations or substitutions such as G1269A (Shaw et al., 2011; Doebele et al., 2012). Second-generation TKIs, including ceritinib, were developed to target mutations such as L1196M, G1269A, I1171T, and S1206Y to overcome this resistance. Despite these advancements, novel mutations continue to confer resistance, necessitating the development of more effective therapeutic options (Friboulet et al., 2014). Third-generation TKIs, such as alectinib, brigatinib, and lorlatinib, have demonstrated improved efficacy against resistant ALK variants, with lorlatinib being the most recently TKI approved by the FDA (Wang et al., 2022) (See Supplementary Figure 1).

A significant challenge in treating ALK-positive NSCLC is the extensive mutational variability of the *eml4-alk* fusion gene and the diversity of amino acid substitutions within the tyrosine kinase domain of the ALK protein (Elshatlawy et al., 2023). However, these mutations may also be present in other types of cancer. Importantly, not all amino acid substitutions act as oncogenic drivers; some are merely passenger mutations or variants of unknown significance (Li et al., 2017). To predict the functional impact of these mutations, computational algorithms such as SIFT (Sorting Intolerant From Tolerant) and PolyPhen-2 (Polymorphism Phenotyping v2) have been developed (Li et al., 2017; Flanagan et al., 2010). SIFT predicts whether an amino acid substitution affects protein function, with scores between 0 and 0.05 indicating deleterious mutations (Ng and Henikoff, 2003). Similarly, PolyPhen-2 assesses the potential impact of amino acid substitutions on protein structure and function, with scores between 0.85 and 1 classified as damaging (Adzhubei et al., 2013).

Given this context, this research aims to analyze data from the Cancer Genome Atlas (TCGA) to identify novel amino acid substitutions within the ALK tyrosine kinase domain that may contribute to oncogenesis. Furthermore, we evaluate the *in silico* binding potential of lorlatinib against these amino acid substitutions to assess its pharmacological sensitivity and explore its possible therapeutic repurpose in other types of cancer.

## Materials and methods

### Collection of *alk* gene somatic mutations

We retrieve primary data from the Cancer Genome Atlas (TCGA) through its official portal https://www.cancer.gov/ccg/research/genome-sequencing/tcga (Center for Cancer Genomics at the National Cancer Institute, 2006). Using the search engine, we queried the *alk* gene using the search engine and extracted all relevant mutation data. The database was then filtered to include only mutations classified as “deleterious” by SIFT and “damaging” by PolyPhen-2. Additionally, we focused on amino acid positions between 1,117 and 1,392, corresponding to the tyrosine kinase domain of the ALK protein (Huang, 2018). As a result, 53 somatic mutations were identified.

### ALK amino acid substitution structure preparation

The structure of the ALK protein in PDB format was obtained from AlphaFold (https://alphafold.ebi.ac.uk/) and identified as AF-Q9UM73-F1-v4. This structure was refined using PyMOL (academic version) (Rigsby and Parker, 2016), focusing on the tyrosine kinase domain. The range of amino acid residues was expanded (from 1,090 to 1,400 residues), and modifications included removing water molecules, adding hydrogen atoms, and incorporating each somatic mutation identified in the TCGA database through mutagenesis. Finally, energy minimization was performed using Swiss PDB Viewer, and all 53 resulting structures were saved in PDB format.

### Obtaining ALK inhibitor ligand

The lorlatinib ligand was retrieved from DrugBank (https://go.drugbank.com/) in PDB format for molecular docking and SMILES format for ADME (absorption, distribution, metabolism, and excretion) analysis using SwisssADME (http://www.swissadme.ch/).

### Molecular docking of ALK amino acid substitution structures with lorlatinib

Each ALK amino acid substitution structure was uploaded into PyRx - Virtual Screening Tool^®^ (free version) to serve as the receptor (Dallakyan and Olson, 2015). The lorlatinib ligand was imported into PyRx using the Open Babel extension and underwent energy minimization. Molecular docking simulations were performed using the AutoDock Vina extension within PyRx, with the grid box parameters set to X = −4.01, Y = −1.59, and Z = −24.75 in all 53 ALK-mutated receptors. Finally, wild-type (WT), C1156Y, and L1196M ALK mutations docked with lorlatinib as a reference group were validated by measuring the root mean square deviation “RMSD” (rmsd reference: <2Å) (Mishra and Sharma, 2016), against crystal structures available in the PDB-RSCB repository, including 7R7R, 5A9U, and 4CLJ, respectively.

### Binding energy measurements

Binding energy is a key parameter that reflects the affinity of a ligand for its binding site on substrate, with more negative values indicating stronger interactions. This study measured the binding energy (in kilocalories per mol, kcal/mol) for the ligand lorlatinib against each previously prepared ALK mutated structure. Binding energies threshold (−6.8 kcal/mol) were calculated by molecular docking of three PDB-RSCB repository: 7R7R, 5A9U, 4CLJ, and AlphaFold model with ATP ligand retrieved from pubchem (see Supplementary Table 1) (NIH, 2025). Molecular interactions were visualized using Discovery Studio Visualizer (free software) to interpret better the binding behavior (Dassault Systemes, 2019).

### Characterization of the protein-ligand interaction profile

Each of the 53 ALK somatic mutation proteins complexed with lorlatinib was saved in pdb format and submitted to the Protein-Ligand Interaction Profiler (PLIP) tool (https://plip-tool.biotec.tu-dresden.de/plip-web/plip/index) (Adasme et al., 2021). The resulting interaction profiles, which classified hydrophobic, hydrogen, and halogen bonds, were downloaded as. pse files, analyzed, and visualized using Pymol.

### Statistical analysis

All data were analyzed and charted in GraphPad Prism 10.03, SRplot (https://www.bioinformatics.com.cn/en) (Tang et al., 2023), PyMOL (Rigsby and Parker, 2016), and BIOVIA Discovery Studio Visualizer software (Dassault Systemes, 2019). Binding energies were performed using the AutoDock Vina parameters (Trott and Olson, 2010). Normality testing and nonparametric analysis were performed using the Kolmogorov-Smirnov test to identify statistically significant changes. Finally, the predictive SIFT and PolyPhen-2 scores were correlated with binding energy values using the Spearman correlation test.

## Results

### Missense mutations in the anaplastic lymphoma kinase *(ALK)* gene encodes for heterogeneous amino acids substitutions associated with different types of cancer

Given the diversity of point mutations in the ALK oncoprotein that contribute to therapy resistance, we explore the TCGA database. A total of 548 somatic mutations were identified, leading to 341 unique missense protein expressions. Of these, 137 mutations were predicted to be deleterious or damaging in tumorigenesis based on SIFT and PolyPhen-2 scores. Specifically, 53 mutations were located within the tyrosine kinase domain (residues 1,090–1,400) (See Supplementary Figure 2).

The most frequently affected position was 1,174, where phenylalanine was substituted by leucine, cysteine, or isoleucine (n = 4; 7.55%). Other recurrent mutations included position 1,202 (n = 2; 3.77%), where glycine was replaced by glutamic acid or arginine, position 1,209 (n = 2; 3.77%), where arginine was substituted by proline or glutamine, and position 1,212, where arginine was replaced by histidine or cysteine. The remaining point mutations were observed only once (n = 1; 1.89%).

These 53 ALK variants were identified in 77 patients (40 males, 37 females), with the highest frequency in corpus uteri (15.58%), followed by adrenal gland (11.69%), skin (10.39%), lung (9.09%), and colon cancer (9.09%). Some mutations were associated with multiple cancer types, such as P1357H (uterus and stomach), R1120W (uterus and colon), R1181H (colon and cervix), G1121D (colon and breast), R1275Q (lymph node and adrenal gland), F1174L (peritoneum, adrenal gland, and others), and F1174C (kidney and adrenal gland) (See Supplementary Tables 2, 3).

**FIGURE 1:**
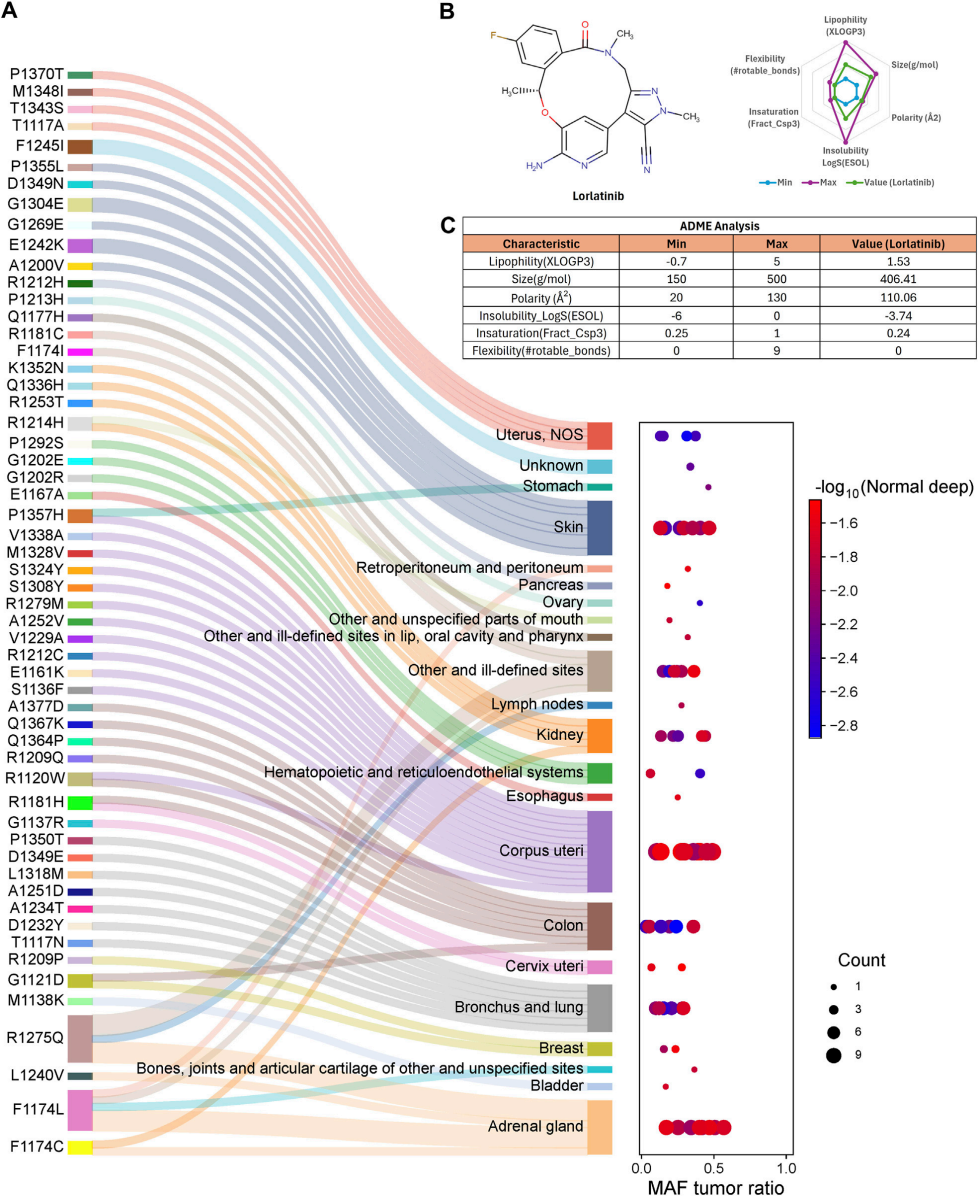
ALK amino acid substitutions are involved in different cancer types. **(A)** Each ALK amino acid substitution is associated with known and newly identified cancer types. Some variants, such as P1357H, G1121D, R1275Q, and F1174 L/C, are implicated in multiple cancer types. The mutant allele frequency (MAF) ratio ranges from 0.034 to 0.568, indicating that these substitutions are rare alleles in the population, **(B)** The Lorlatinib molecule meets the criteria for six key ADME (absorption, distribution, metabolism, and excretion) parameters, supporting its pharmacokinetic suitability, **(C)** Table summarizing the specific values for the six ADME parameters of lorlatinib.

In general, these ALK variants are associated with multiple cancer types. All 53 ALK amino acid substitutions had tumor mutant allele frequency (MAF) values ranging from 0.034 to 0.568, classifying them as rare or less frequent alleles in the population, in the context of TCGA data, MAF or also known as “variant allele frequency” (VAF) refers to the proportion of reads at a specific genomic location that carry a somatic mutation, indicating the percentage of tumour cells harbouring that mutation. Despite their low frequency, all 53 ALK variants were predicted to be deleterious or damaging according to SIFT and PolyPhen-2 scores (Figure 1A). These findings suggest that ALK could be a therapeutic target for cancers beyond non-small cell lung cancer (NSCLC). Notably, lorlatinib, the latest FDA-approved ALK inhibitor for NSCLC (The Asco Post, 2018), has demonstrated favorable pharmacokinetics properties, including ADME (absorption, distribution, metabolism, and excretion) parameters. These properties support its potential repurpose as a therapeutic option for other ALK-driven malignancies (See Figures 1B,C; Supplementary Table 4).

### Novel somatic variants of the ALK structure exhibit strong binding energy to lorlatinib

**FIGURE 2:**
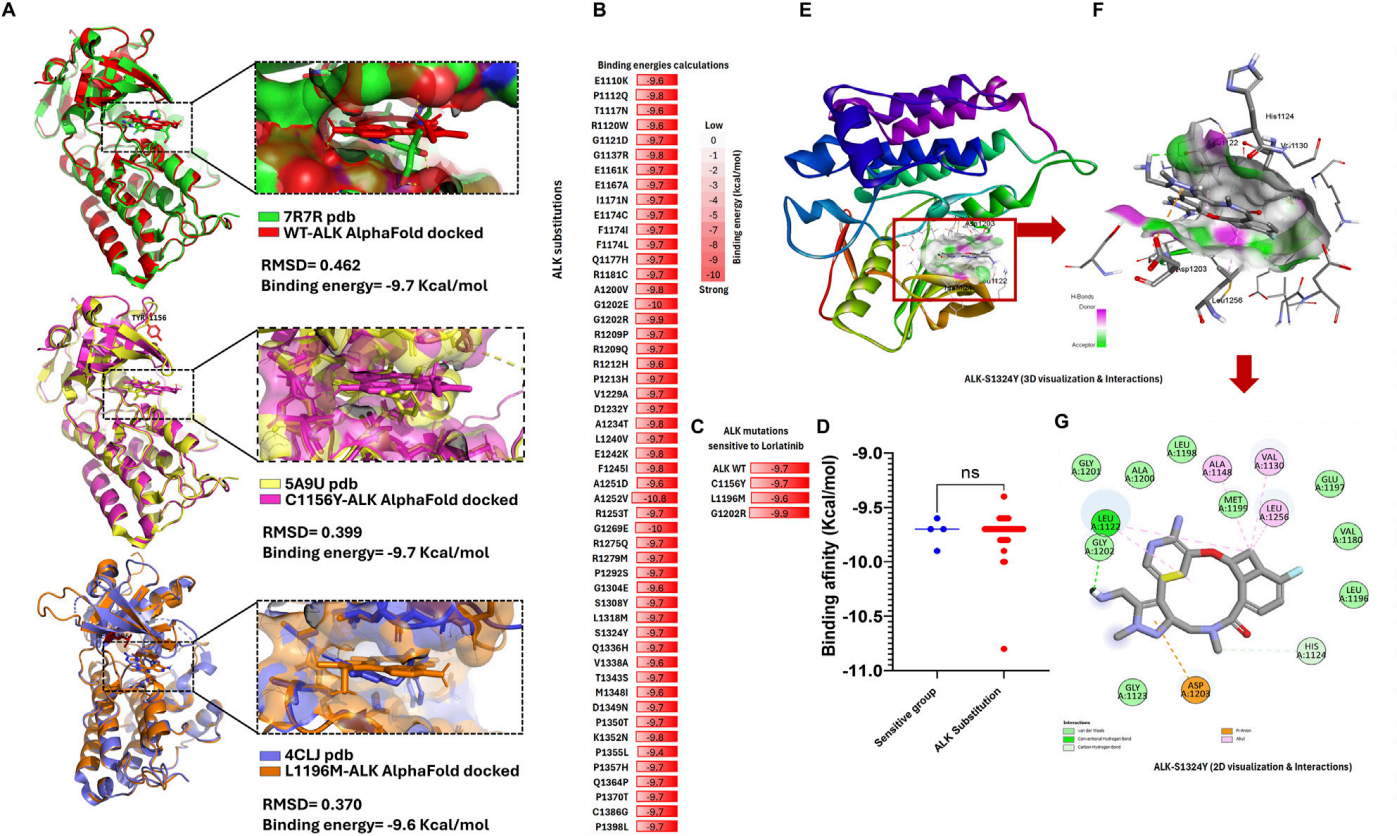
ALK structure variants exhibit strong binding energy to lorlatinib comparable to the know sensitive group **(A)** We can see the validation structure obtained from AlphaFold with crystallized structure available in RSCB-PDB, here we can corroborate that alphaFold structure displays RMSD values close to zero (reference <2 Å). **(B)** List of ALK amino acids substitutions, highlighting in red boxes containing their respective binding energy values calculated by AutoDock Vina in kcal/mol, **(C)** List of reference ALK protein group: WT, C1156Y, L1196M, and G1202R sensitive to lorlatinib, biding energy values (contained in red boxes) calculated by AutoDock Vina are used as control binding energy, **(D)** Nonparametric T-test analysis comparing the binding energies of the 53 ALK variants versus the ALK-sensitive reference group, showing no significant differences (ns), **(E)** Tridimensional structure of the S1324Y-lorlatinib interaction, highlighting key binding residues, **(F)** Zoom-in view of the S1324Y-lorlatinib interaction, highlighting key binding residue, **(G)** 2D schematic representation of the S1324Y-lorlatinib binding interactions.

Lorlatinib is the latest FDA-approved ALK inhibitor designed to overcome resistance in non-small cell lung cancer (NSCLC). It effectively targets mutations such as C1156Y, I1171 N/S/T, L1196M, and G1202R, which confer resistance to earlier-generation ALK inhibitors (Trott and Olson, 2010). We selected key ALK mutations sensitive to lorlatinib to establish a reference group, such as wild-type (WT), C1156Y, L1196M, and G1202R. This group, termed the “ALK protein group sensitive to lorlatinib,” was used for comparative analysis. Structural data for three of them were obtained from the Protein Data Bank (PDB-RSCB, https://www.rcsb.org/), including: 7R7R (ALK-WT-lorlatinib complex), 5A9U (C1156Y-lorlatinib complex), and 4CLJ (L1196M-lorlatinib complex) (Figure 2A).

Given that lorlatinib binds effectively to the ALK protein, we hypothesized that it could also interact with all 53 newly identified ALK variants. However, its efficacy against these novel mutations remained unknown. To address this, we performed molecular docking experiments using ALK protein structures obtained from AlphaFold, introducing the 53 somatic mutations as well as the reference mutations, followed by lorlatinib binding simulations (Figure 2B).

Our results demonstrated that the binding energy of lorlatinib for the ALK-sensitive reference group was: −9.6 kcal/mol (L1196M), −9.7 kcal/mol (ALK wild-type and C1156Y), and −9.9 kcal/mol (G1202R). Additionally, validation against available crystal structures (RSCB-PDB IDs: 7R7R, 5A9U, and 4CLJ) resulted in RMSD values of 0.462, 0.399, and 0.370, respectively, confirming that the docking models closely resemble experimentally determined structures. These findings suggest that strong binding energy correlates with sensitivity to lorlatinib (Figure 2C). Surprisingly, all 53 ALK variants also exhibited strong binding energy, ranging from −9.4 kcal/mol (P1355L) to −10.8 kcal/mol (A1252V) (AutoDock Vina analysis), indicating that lorlatinib maintains a high affinity for these mutations. Moreover, a nonparametric T-test comparison between the 53 ALK variants and the ALK-sensitive reference group showed no significant differences in binding energy (Figure 2D).

These results suggest that lorlatinib could effectively bind to and inhibit these 53 novel ALK somatic mutations, potentially regulating ALK signaling in multiple cancer types beyond NSCLC. The key amino acid residues mediating lorlatinib interactions will be described in the following section (Figures 2D–G).

### Lorlatinib binds to ALK variants through strong hydrophobic, hydrogen, and halogen bond interactions

**FIGURE 3:**
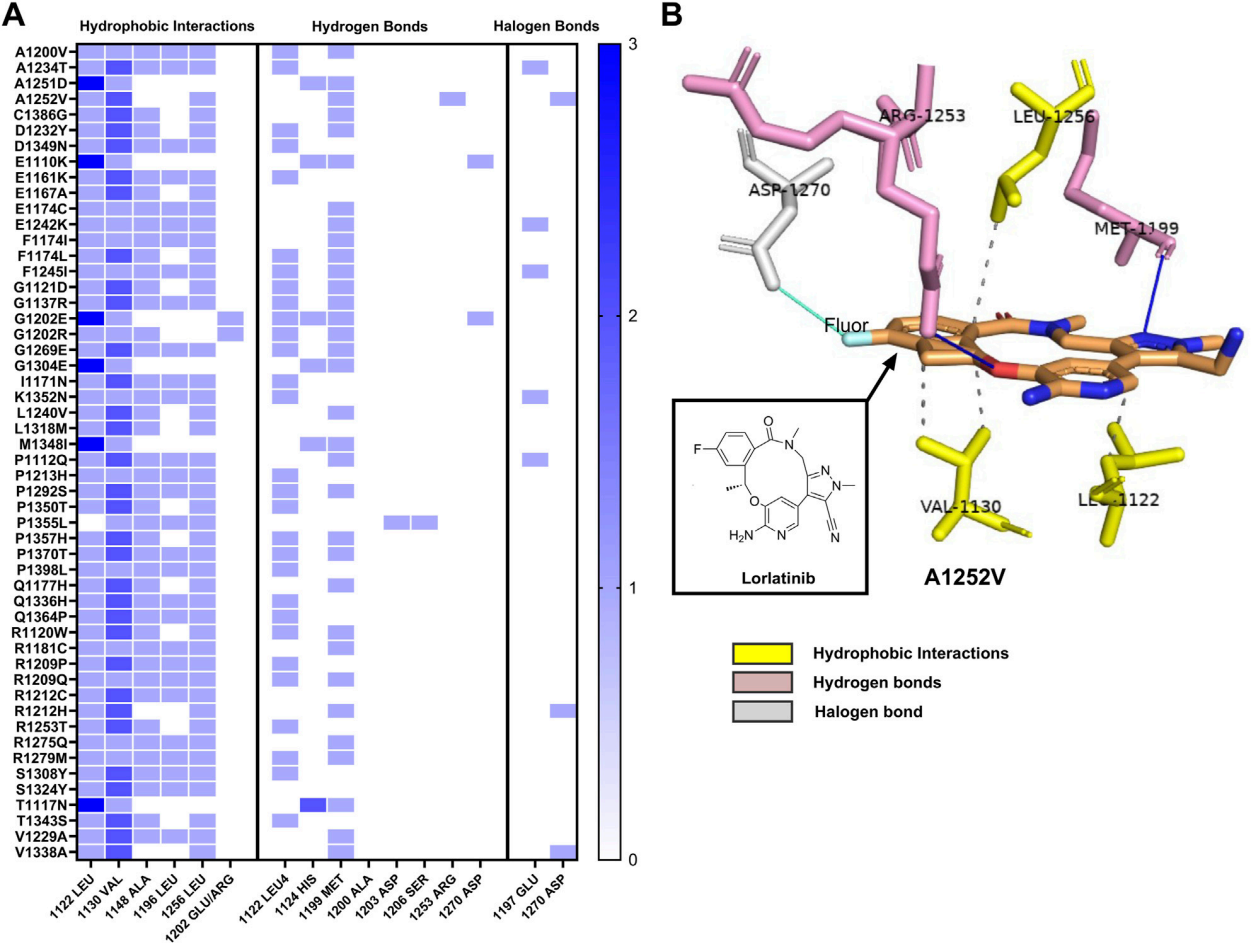
Interaction profile of ALK variants with lorlatinib by hydrophobic interaction, hydrogen, and halogen bonds **(A)** Heatmap illustrating the three main types of interactions between shows three groups of interactions of ALK structure variants with lorlatinib: hydrophobic interaction, hydrogen and halogen bonds, each blue box displays interactions frequency with key amino acids (from 0 to 3), **(B)** Representative three-dimensional structure of the ALK variants S1252V, highlighting its interactions with lorlatinib. The image displays hydrophobic interactions in discontinuous lines with Leu1256, Val1130 and Leu1122 (amino acids in yellow), and hydrogen bonds interactions represented in blue continuous lines with Arg1253 and Met1199 (amino acids in pink), and halogen bond interaction represented in cyan continuous line with Asp1270 amino acid in gray color, demonstrating the multifaceted binding mechanism that stabilizes lorlatinib within the active site of the ALK protein.

Molecular docking analysis revealed that each ALK variant exhibited a specific interaction pattern with lorlatinib, categorized into three main types: hydrophobic interactions, hydrogen bonds, and halogen bonds. These interactions play a crucial role in stabilizing the ligand within the protein’s active site and may contribute to the drug’s efficacy against novel *ALK* mutations (Figures 3A,B).

Hydrophobic interactions were the most prevalent type of interaction observed across the analyzed variants. Notably, valine at position 1,130 was involved in hydrophobic interactions in all ALK variants (100%), followed by leucine at positions 1,122 and 1,256, which were present in 98% and 84.9% of cases, respectively. Additionally, alanine at position 1,148 contributed to these interactions in 81.1% of the variants, while leucine at position 1,196 was involved in 54.7% of cases. Interesting, two substitutions at position 1,202 (glutamic acid and arginine) also maintained hydrophobic interactions with lorlatinib, suggesting that the overall binding environment of this region remains favorable for drug engagement despite the presence of mutations.

Hydrogen bonds, another key interaction type, were primarily observed at methionine 1,199, which formed hydrogen bonds in 62.3% of the variants. Additionally, leucine 1,122 participated in hydrogen bonding in 54.7% of cases, reinforcing its critical role in lorlatinib binding. Less frequently observed hydrogen bond interactions included histidine 1,124 (six cases), aspartate 1,270 (two cases), aspartate 1,203 (one case), serine 1,206 (one case), and arginine 1,253 (one case). These interactions suggest that lorlatinib maintains a stable binding conformation across a broad range of *ALK* variants, which could explain the strong binding affinity observed in molecular docking simulations.

In addition to hydrophobic and hydrogen bond interactions, sole ALK variants could also form halogen bonds with lorlatinib due to its fluorine atom. These halogen bonds were primarily established with glutamine at position 1,197, detected in variants A1234T, E1242K, F1245I, K1352N, and P1112Q. Additionally, aspartate at position 1,270 was involved in halogen bonding interactions in A1252V, R1212H, and V1338A. The ability of lorlatinib to form these halogen bonds may further contribute to its binding stability and potential inhibitory effects on mutated ALK proteins.

Taken together, these findings suggest that lorlatinib establishes a robust binding network across the analyzed ALK variants. In particular, leucine at position 1,122 appears to be a key residue, participating in both hydrophobic and hydrogen-bond interactions in most ALK variants.

This could play a critical role in maintaining drug sensitivity, even in the presence of somatic mutations classified as deleterious or damaging by tumor predictor algorithms such as SIFT and PolyPhen-2. These results provide valuable insights into the molecular basis of lorlatinib’s interaction with novel *ALK* mutations and support its potential therapeutic relevance in a broader range of cancer types beyond non-small cell lung cancer (NSCLC).

### The discrepancy between binding affinity scores of ALK variants and somatic mutation predictor scores

**FIGURE 4:**
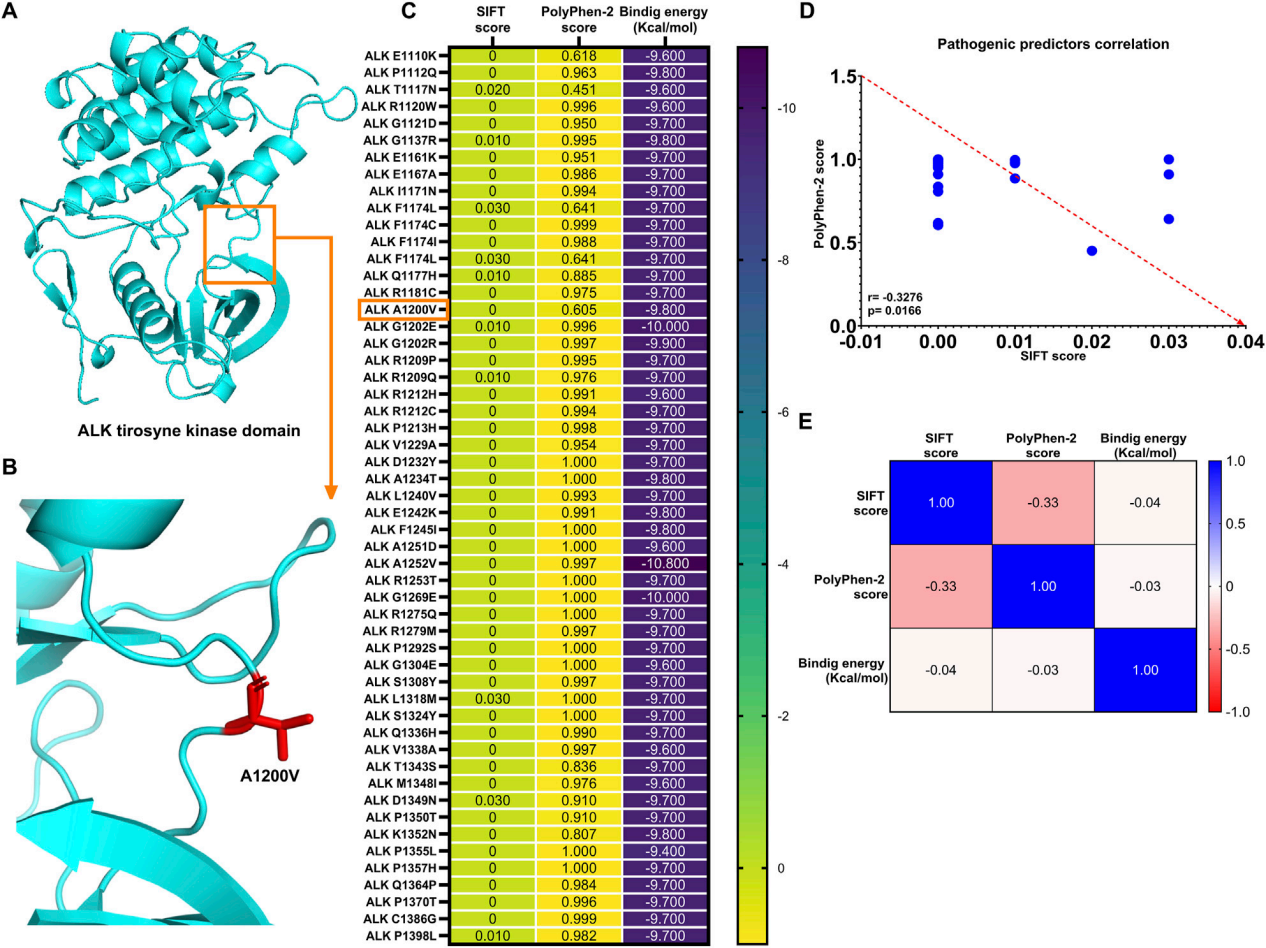
ALK structure variants exhibit strong binding energy to lorlatinib as known sensitive group **(A)** ALK protein structure obtained from AlphaFold visualized using PyMOL (academic version), **(B)** ALK variant, A1200V, generated through mutagenesis in PyMOL, with substituted residue highlighted in red stick, **(C)** Heatmap displaying SIFT and PolyPhen-2 scores for all 53 ALK variants implicated in different cancer types, along with their respective binding (Kcal/mol), **(D)** Spearman’s correlation analysis reveals a significant negative correlation between SIFT and PolyPhen-2 score, and **(E)** Tumorigenic predictor scores (SIFT and PolyPhen-2) show not significant correlation with binding energies of the 53 ALK amino acids substitutions.

All 53 ALK variants were manually generated using PyMOL academic software, following the methodology described in the Methods section. The structural modifications were based on the AlphaFold-derived ALK tyrosine kinase domain template (Figures 4A,B). Two computational predictors of tumorigenicity were used: SIFT, where a score close to or equal to 0 classifies substitutions as deleterious (indicating potential disruption of protein function), and PolyPhen-2, where a score close to or equal to 1 classified a substitution as damaging (suggesting structural and functional impairment of the protein). Additionally, molecular docking analyses using AutoDock Vina revealed binding energy values ranging from −9.6 to −10.8 kcal/mol (Figure 4C).

The expected inverse correlation between SIFT and PolyPhen-2 scores was confirmed by Spearman’s correlation test (r = −0.3276, p = 0.0166), indicating a statistically significant negative association between these predictive metrics (Figure 4D). However, when comparing these tumorigenic predictor scores with binding energy values, the correlations were notably weak (r SIFT vs. binding energy = −0.04; r PolyPhen-2 vs. binding energy = −0.03) and statistically nonsignificant (p = 0.791 and p = 0.818, respectively) (Figure 4E).

These results suggest the relationship between computational predictors of oncogenic potential and molecular properties. Although SIFT and PolyPhen-2 scores predicted that these ALK substitutions could contribute to tumorigenesis, the strong binding affinity of lorlatinib indicates its potential to effectively target these ALK variants, possibly mitigating their oncogenic effects (See Supplementary 4).

## Discussion

Amino acid point mutations in the ALK protein are critical in conferring resistance to ALK inhibitor therapies in cancer. Notably, mutations such as G2032R and I1151Tins are associated with resistance to the first-generation inhibitor crizotinib. Conversely, mutations such as C1156T/Y, L1198F, D1203N, and G1202R have been linked to resistance against second-generation inhibitors, including ceritinib, alectinib, and brigatinib. Furthermore, third-generation inhibitors like lorlatinib exhibit resistance to mutations such as L1198F, G2032R, D1203N, and G1123D (Poei et al., 2024). Despite these challenges, lorlatinib has demonstrated significant therapeutic efficacy in overcoming drug resistance (Poei et al., 2024; Johnson et al., 2014).

In this study, we identified 53 amino acid substitutions in the ALK protein using the ATCG database. We classified them as potential oncogenic drivers based on predictive tools such as SIFT and PolyPhen-2 scores. These mutations have been associated with various cancers, including NSCLC, anaplastic large-cell lymphoma, neuroblastoma, and colorectal carcinoma. Moreover, our findings suggest novel associations of these mutations with additional cancer types, including skin, retroperitoneal, peritoneal, pancreatic, kidney, esophageal, breast, bladder, and adrenal tumors (Holla et al., 2017; Adzhubei et al., 2010; Bresler et al., 2014).

Furthermore, lorlatinib has demonstrated the ability to target multiple ALK mutations, including C1156Y, I1171 N/S, F1174C, L1196M, L1198F, G1202R, D1203N, E1210K, and G1269A, showcasing sensitivity in NSCLC patients, even those resistant to prior-generation ALK inhibitors. This suggests that lorlatinib may potentially address a broader spectrum of ALK amino acid substitutions, positioning it as a promising candidate for repurposing therapies in various types of cancer (Bertacca et al., 2023). Our hypothesis was further validated by binding energy calculations for all 53 ALK variants revealed values comparable to those of experimentally validated ALK wild-type (WT), C1156Y, L1196M, and G1202R mutations, which are known to be sensitive to lorlatinib (Poei et al., 2024; Gainor et al., 2016).

Among the 53 identified ALK amino acid substitutions, three have been reported to interact with the active site of ALK protein. We identified two structures containing the F1174L mutation (PDB codes: 2YJR and 4FNW) and four structures for R1275Q (PDB codes: 4FNY, 4FNX, 4FNZ, and 4FNW), both implicated in neuroblastoma (Epstein et al., 2012; Sasaki et al., 2010). Additionally, a structure harboring the G1202R mutation (PDB code: 9GBE) was identified in complex with the NVL-655 ALK inhibitor for NSCLC, suggesting that lorlatinib may also target this variant (Lin et al., 2024). Furthermore, a case report in NSCLC demonstrated that the ALK I1171N mutation, along with other ALK amino acids substitutions identified in this study, conferred resistance to ensartinib but remained sensitive to lorlatinib, further supporting the efficacy of lorlatinib against multiple ALK amino acids substitutions (Ye and Guo, 2023). The remaining 49 ALK amino acids substitutions may also contribute to oncogenesis, as their oncogenic predictor scores suggest potential alterations in the ALK protein. Although these mutations have not yet been documented in the literature, they are available in the TCGA database and have been associated with different types of cancer, with some mutations occurring in more than one cancer type.

Given that lorlatinib has demonstrated efficacy against multiple ALK amino acids substitutions, our results suggest that it could target all 53 identified substitutions, as indicated by binding energy calculations and protein-ligand interaction profiles. Previous studies have reported binding energy values of −8.9, −8.6, and −8.4 kcal/mol for ALK-WT, F1174C, and F1174L, respectively, when interacting with lorlatinib. In contrast, our results for the same mutations yielded a stronger binding energy of −9.7 kcal/mol. Similarly, binding energy values ranging from −9.6 to −10.8 kcal/mol were observed for the remaining ALK amino acid substitutions analyzed in this study (Balasundaram and Doss, 2023), further supporting lorlatinib’s potential as a versatile therapeutic option.

The strong binding energy affinities observed for all 53 ALK amino acid substitutions can be attributed to key stabilizing interactions, including hydrophobic and hydrogen bond interactions, so they play an important role because they stabilize the protein-ligand energy (Varma et al., 2010). Specifically, lorlatinib exhibited strong hydrophobic interactions with Leu 1,122, Val 1,130, Ala 1,148, Leu 1,196, and Leu 1,256, as well as hydrogen bonds with Leu 1,122 and Met 1,199. Additionally, halogen bonds with Glu 1,197 and ASP 1270 were identified for substitutions lacking hydrogen bonds, which are critical in drug design as they enhance protein-ligand interactions. Fluorine and chlorine atoms are frequently incorporated into drug structures to improve physicochemical properties (Shinada et al., 2019; Margiotta et al., 2020), which may explain why ALK amino acid substitutions such as A1234T, A1252V, E1242K, F1245I, K1352N, P1112Q, R1212H, and V1338A exhibit strong binding to lorlatinib, as its molecular conformation includes a fluorine atom.

In the TCGA database, 541 somatic ALK amino acid substitutions were identified; however, not all were predicted to be oncogenic drivers. Only 53 were classified as tumorigenic based on SIFT and Polyphen-2 predictor scores within the ALK tyrosine kinase domain. These predictors, which range from 0 to 1, assess the likelihood that an amino acid substitution will impact protein function. SIFT scores between 0 and 0.05 indicate deleterious effects, while PolyPhen-2 scores from 0.85 to 1 classify substitutions as damaging (Ng and Henikoff, 2003; Adzhubei et al., 2013). In our analysis, all 53 ALK amino acid substitutions exhibited SIFT scores close to 0 and PolyPhen-2 scores near 1, confirming their deleterious and damaging nature, respectively. Previous studies have proposed a combined model using both predictors to assess non-synonymous variants, showing a positive correlation between 1-SIFT scores and PolyPhen-2 scores (Wei et al., 2011). However, in our study, we observed a negative correlation, which may be attributed to the direct comparison of PolyPhen-2 scores with the original SIFT scores rather than their complementary 1-SIFT values.

While the SIFT and PolyPhen-2 predictors did not exhibit a strong correlation with binding energy, our analysis revealed a weak negative correlation (See Figure 4E), likely due to random variations. Nevertheless, the binding energies to lorlatinib suggest that lorlatinib remains effective against all 53 ALK amino acid substitutions classified as deleterious or damaging in cancer. These findings support the potential repurpose of lorlatinib to target additional ALK-driven cancers beyond NSCLC, as indicated by *in silico* simulations.

In this study, we acknowledge a limitation in relying solely on *in silico* tools such as SIFT and PolyPhen-2 for assessing the oncogenic potential of ALK genomic alterations. While these tools offer valuable predictive insights into variant pathogenicity, they do not encompass the full spectrum of clinical oncogenicity criteria. Future research should explore the broader context of ALK interactions and functional consequences to enhance understanding of their oncogenic roles.

To verify the predictive potential of pathogenicity, we conducted a manual search on the Alphamissense portal: https://alphamissense.hegelab.org/search (Author anonymous, 2025; Minton, 2023). Through this approach, we confirmed that all the 53 genetic variants we proposed were classified as likely pathogenic, with the majority exceeding a confidence score of 0.89 (See Supplementary Table 5). Additionally, we evaluated the amino acid changes associated with oncogenicity or treatment resistance using the OncoKB™ database (Suehnholz et al., 2024; Chakravarty et al., 2017), which is one of the most reputable platforms for genetic variant curation, adhering to rigorous standards of somatic variants classification (Horak et al., 2022).

On the other hand, while the exact impact on ATP-binding or kinase activation for these specific novel substitutions requires experimental validation, their classification as “likely pathogenic” suggests they may contribute to aberrant ALK signaling. Several identified mutations have known biological and clinical implications according to OncoKB™ (https://www.oncokb.org/gene/ALK) (Chakravarty et al., 2017; ALK, 2017). The ALK I1171N (p.Ile1171Asn) mutation is classified as “Likely Oncogenic” and is clinically relevant due to its resistance to first- and second-generation ALK inhibitors (crizotinib, ceritinib, and alectinib), while notably retaining sensitivity to brigatinib and lorlatinib (ALK, 2017; Ceccon et al., 2013). This highlights a critical mechanism of acquired drug resistance. Similarly, ALK F1174L (p.Phe1174Leu) is an “Oncogenic” mutation associated with resistance to crizotinib, ceritinib, and alectinib, but shows sensitivity to lorlatinib and brigatinib. The ALK F1174C (p.Phe1174Cys) mutation, also “Likely Oncogenic,” exhibits resistance to crizotinib and ceritinib but remains sensitive to alectinib, brigatinib, and lorlatinib. Conversely, ALK F1174I (p.Phe1174Ile), another “Likely Oncogenic” variant, displays a broader sensitivity to crizotinib, ceritinib, alectinib, and lorlatinib. These F1174 variants underscore the diverse therapeutic challenges and opportunities within a single amino acid position (see Supplementary 7) (ALK, 2017; Cheng et al., 2023).

Furthermore, the ALK G1202R (p.Gly1202Arg) mutation is categorized as a “Resistance” mutation, confirming its role in resistance to crizotinib, ceritinib, alectinib, and brigatinib, while being sensitive to lorlatinib. This well-characterized mutation is a key driver of acquired resistance to several ALK TKIs. In contrast, the ALK A1200V (p.Ala1200Val) and ALK E1242K (p.Glu1242Lys) mutations are both deemed “Likely Neutral” by OncoKB, suggesting they may not significantly impact ALK function or drug response. Lastly, the ALK R1275Q (p.Arg1275Gln) mutation is identified as “Oncogenic” and has demonstrated sensitivity to crizotinib and lorlatinib in both *in vitro* and *in vivo* studies, indicating its potential as a targetable alteration (see Supplementary 7) (ALK, 2017; Cheng et al., 2023).

The consistent sensitivity of several ALK mutations demonstrated in our *in silico* experiments, including I1171N, F1174L, F1174C, F1174I, G1202R, and R1275Q, to lorlatinib, as highlighted by our findings and supported by OncoKB classifications, strongly suggests the potential for therapeutic repurposing of this third-generation ALK inhibitor beyond its current primary indication in NSCLC. Lorlatinib’s known ability to overcome common resistance mutations that emerge from earlier-generation ALK TKIs, coupled with its excellent central nervous system (CNS) penetration, makes it a highly promising candidate for other ALK-driven malignancies, particularly those with a propensity for CNS metastases, such as neuroblastoma. This work influences future preclinical and clinical investigations by providing a rationale to explore lorlatinib’s efficacy in a broader spectrum of ALK-driven cancers.

Future steps should include: (Huret and Dessen, 2024): Preclinical validation: detailed *in vitro* and *in vivo* studies in diverse ALK-driven cancer models (e.g., specific lymphomas, inflammatory myofibroblastic tumors, or other solid tumors where ALK fusions or activating mutations are identified) harboring these specific sensitive mutations, to thoroughly characterize its anti-tumor activity and optimal dosing; (Yao et al., 2013); Biomarker-driven clinical trials: initiating basket or umbrella clinical trials specifically enriching for patients with these lorlatinib-sensitive ALK mutations, regardless of cancer type, to evaluate clinical response rates and safety in a real-world setting; and (Holla et al., 2017) Investigation of combination therapies: exploring rational combinations of lorlatinib with other targeted agents or conventional therapies to potentially overcome emergent resistance mechanisms or enhance therapeutic efficacy. By leveraging the comprehensive genomic and functional data presented here, a more precise and personalized approach to treating ALK-driven cancers can be developed, potentially improving patient outcomes in a wider range of malignancies.

In conclusion, our study identified novel amino acid substitutions in the ALK tyrosine kinase domain associated with cancers beyond hematological malignancies and NSCLC. Importantly, we demonstrated that lorlatinib retains efficacy against these mutations, suggesting its potential for therapeutic repurposing in other ALK-mutated cancers. This finding persists despite SIFT and PolyPhen-2 predictions of oncogenic progression, positioning lorlatinib as a promising candidate for broader clinical applications.

## Data Availability

The original contributions presented in the study are included in the article/Supplementary Material, further inquiries can be directed to the corresponding author.

## Supplementary material

SUPPLEMENTARY FIGURE 1 Mechanism of action of lorlatinib in tumoral cell. The ALK gene, located on the short arm of chromosome 2, encodes the transmembrane ALK protein. In contrast, the EML4-ALK fusion gene produces an oncogenic ALK variant that lacks the extracellular and transmembrane domains, retaining only the intracellular region containing the tyrosine kinase domain. This truncated protein becomes susceptible to constitutive phosphorylation by ATP, leading to dysregulation of signaling pathways involved in cell proliferation and survival. Lorlatinib, a small-molecule inhibitor, crosses the cell membrane and competes with ATP for binding at ALK’s active site, thereby modulating these signaling pathways.

SUPPLEMENTARY FIGURE 2 Workflow data obtaining This is the workflow followed to obtain our data. Here, 548 ALK variants were available on TCGA, of which 341 displayed missense mutations. Only 137 variants were characterized as deleterious/damaging as predicted by SIFT and PolyPhen algorithms. Finally, we focused on 53 ALK substitutions that affected the tyrosine kinase domain.
