# Supplementary material for: An in silico evaluation of lorlatinib as a potential therapy for novel amino acid substitutions in the tyrosine kinase domain of the ALK protein associated with cancer
Source: Front Pharmacol. 2025 Jun 18;16:1605314. doi: 10.3389/fphar.2025.1605314 (PMC12213726; doi:10.3389/fphar.2025.1605314)
Supplement: Supplementary file 1 [file Supplementaryfile1.docx]

**Supplementary information**

**Supplementary figure 1: Mechanism of action of lorlatinib in tumoral cell**

The ALK gene, located on the short arm of chromosome 2, encodes the transmembrane ALK protein. In contrast, the EML4-ALK fusion gene produces an oncogenic ALK variant that lacks the extracellular and transmembrane domains, retaining only the intracellular region containing the tyrosine kinase domain. This truncated protein becomes susceptible to constitutive phosphorylation by ATP, leading to dysregulation of signaling pathways involved in cell proliferation and survival. Lorlatinib, a small-molecule inhibitor, crosses the cell membrane and competes with ATP for binding at ALK's active site, thereby modulating these signaling pathways

**Supplementary figure 2: Workflow data obtaining**

This is the workflow followed to obtain our data, here 548 ALK variants were available on TCGA, where 341 variants displayed missense mutation and only 137 variants were characterized as deleterious/damaging predicted by SIFT and Polyphen algorithms, then finally we focused on 53 ALK substitutions that affected tyrosine kinase domain.

**Supplementary table 1: Threshold binding energy calculation**

| **PDB Receptor (ALK)** | **Ligand** | **Molecular Docking Grid Box** | **Binding affinity (Kcal/mol)** |
| --- | --- | --- | --- |
| 4CLJ | ATP | x = -38.0498 y = -19.1741 z = 69.1316 | -6.6 |
| 5A9U | ATP | x = -38.1646 y = -19.2154 z = 69.2192 | -7.0 |
| 7R7R | ATP | x = -38.1246 y = -19.3043 z = 69.319 | -7.0 |
| AlphaFold_ALK | ATP | x = -4.0092 y = -1.5922 z = -24.7604 | -6.6 |
| **Threshold binding energy mean** |  |  | **-6.8 ± 0.2** |

To establish the binding energy threshold, we performed molecular docking of three crystallographic structures from the RCSB PDB repository, along with the AlphaFold model, using the constitutive phosphorylation molecule (ATP) as the reference ligand. This analysis yielded an average threshold energy of -6.8 ± 0.2 kcal/mol.

**Supplementary table 2: ALK substitutions data available in TCGA display MAF ratio**

| **ALK substitution** | **Position** | **Primary site** | **Project TCGA ID** | **Gender** | **Age of diagnosis** | **Tumor MAF (Mutant allele frequency)** | **Ratio Tumor MAF (Mutant allele frequency)** | **Normal depth** |
| --- | --- | --- | --- | --- | --- | --- | --- | --- |
| T1117A | 1117 | Uterus, NOS | TCGA-LUAD | Male | 56 | 11/91 | 0.312 | 647 |
| T1343S | 1343 | Uterus, NOS | CPTAC-3 | Female | 85 | 167/535 | 0.373 | 298 |
| M1348I | 1348 | Uterus, NOS | CPTAC-3 | Female | 55 | 162/677 | 0.15 | 268 |
| P1370T | 1370 | Uterus, NOS | CPTAC-3 | Female | 53 | 28/62 | 0.129 | 291 |
| F1245I | 1245 | Unknown | TARGET-NBL | Male | 3 | 11/46 | 0.337 | 204 |
| F1245I | 1245 | Unknown | TARGET-NBL | Male | 3 | 12/78 | 0.337 | 204 |
| P1357H | 1357 | Stomach | TCGA-UCEC | Female |  | 10/34 | 0.462 | 139 |
| A1200V | 1200 | Skin | TCGA-SKCM | Male | 72 | 10/36 | 0.467 | 49 |
| E1242K | 1242 | Skin | TCGA-SKCM | Male | 80 | 6/36 | 0.13 | 49 |
| E1242K | 1242 | Skin | TCGA-SKCM | Male | 53 | 22/45 | 0.352 | 76 |
| G1269E | 1269 | Skin | TCGA-SKCM | Female | 65 | 10/40 | 0.406 | 111 |
| G1304E | 1304 | Skin | TCGA-SKCM | Male | 79 | 18/56 | 0.16 | 264 |
| G1304E | 1304 | Skin | CMI-ASC | Male | 74 | 6/35 | 0.292 | 55 |
| D1349N | 1349 | Skin | HCMI-CMDC | Female | 65 | 12/30 | 0.265 | 228 |
| P1355L | 1355 | Skin | TCGA-SKCM | Male | 68 | 23/49 | 0.291 | 134 |
| F1174L | 1174 | Retroperitoneum and peritoneum | TARGET-NBL | Male | 6 | 15/36 | 0.32 | 37 |
| R1212H | 1212 | Pancreas | TCGA-UCEC | Female | 57 | 27/74 | 0.178 | 30 |
| P1213H | 1213 | Ovary | CPTAC-2 | Female |  | 50/120 | 0.403 | 394 |
| R1214H | 1214 | Other and unspecified parts of mouth | CPTAC-3 | Male | 58 | 16/71 | 0.193 | 57 |
| Q1177H | 1177 | Other and ill-defined sites in lip, oral cavity and pharynx | TCGA-HNSC | Male | 66 | 21/58 | 0.319 | 62 |
| F1174I | 1174 | Other and ill-defined sites | TARGET-NBL | Male | 4 | 16/50 | 0.225 | 54 |
| F1174L | 1174 | Other and ill-defined sites | TARGET-NBL | Female | 8 | 15/47 | 0.362 | 36 |
| R1181C | 1181 | Other and ill-defined sites | TCGA-CESC | Female | 53 | 5/73 | 0.193 | 398 |
| R1275Q | 1275 | Other and ill-defined sites | TARGET-NBL | Female | 1 | 67/500 | 0.149 | 130 |
| R1275Q | 1275 | Other and ill-defined sites | TARGET-NBL | Male | 1 | 76/393 | 0.244 | 90 |
| R1275Q | 1275 | Other and ill-defined sites | TARGET-NBL | Male | 5 | 14/30 | 0.276 | 82 |
| R1275Q | 1275 | Lymph nodes | TARGET-NBL | Male | 15 | 241/593 | 0.276 | 78 |
| F1174C | 1174 | Kidney | TARGET-NBL | Male | 2 | 236/585 | 0.417 | 46 |
| R1214H | 1214 | Kidney | TCGA-HNSC | Male | 73 | 22/114 | 0.252 | 219 |
| R1253T | 1253 | Kidney | TCGA-KIRP | Female | 59 | 16346 | 0.134 | 91 |
| Q1336H | 1336 | Kidney | TCGA-KIRP | Male | 70 | 8/27 | 0.44 | 67 |
| K1352N | 1352 | Kidney | TCGA-KIRC | Female | 63 | 29/163 | 0.218 | 139 |
| G1202E | 1202 | Hematopoietic and reticuloendothelial systems | MP2PRT-ALL | Male | 5 | 142/352 | 0.403 | 358 |
| G1202R | 1202 | Hematopoietic and reticuloendothelial systems | MP2PRT-ALL | Male | 5 | 57/226 | 0.406 | 357 |
| P1292S | 1292 | Hematopoietic and reticuloendothelial systems | MMRF-COMMPASS | Male | 61 | 11/57 | 0.061 | 54 |
| E1167A | 1167 | Esophagus | TCGA-ESCA | Female | 84 | 11/40 | 0.25 | 41 |
| R1120W | 1120 | Corpus uteri | CPTAC-2 | Male |  | 50/327 | 0.452 | 59 |
| S1136F | 1136 | Corpus uteri | TCGA-UCEC | Female | 53 | 7/47 | 0.294 | 43 |
| E1161K | 1161 | Corpus uteri | TCGA-UCEC | Female | 34 | 25/44 | 0.489 | 45 |
| R1212C | 1212 | Corpus uteri | TCGA-PAAD | Male | 71 | 19/54 | 0.296 | 49 |
| V1229A | 1229 | Corpus uteri | TCGA-UCEC | Female | 51 | 10/77 | 0.275 | 33 |
| A1252V | 1252 | Corpus uteri | TCGA-UCEC | Female |  | 61/181 | 0.133 | 34 |
| R1279M | 1279 | Corpus uteri | TCGA-UCEC | Female | 55 | 61/181 | 0.105 | 89 |
| S1308Y | 1308 | Corpus uteri | TCGA-UCEC | Female | 50 | 18/62 | 0.358 | 103 |
| S1324Y | 1324 | Corpus uteri | TCGA-UCEC | Female | 40 | 4/30 | 0.4 | 33 |
| M1328V | 1328 | Corpus uteri | TCGA-UCEC | Female | 34 | 13/97 | 0.406 | 47 |
| V1338A | 1338 | Corpus uteri | TCGA-UCEC | Female | 47 | 28/69 | 0.286 | 44 |
| P1357H | 1357 | Corpus uteri | TCGA-STAD | Male | 84 | 27/79 | 0.388 | 31 |
| R1120W | 1120 | Colon | TCGA-UCEC | Female | 60 | 41/81 | 0.239 | 749 |
| G1121D | 1121 | Colon | TCGA-COAD | Male | 82 | 26/103 | 0.239 | 63 |
| R1181H | 1181 | Colon | CPTAC-2 | Female |  | 24/87 | 0.134 | 321 |
| R1209Q | 1209 | Colon | TCGA-COAD | Male | 57 | 29/119 | 0.193 | 145 |
| Q1364P | 1364 | Colon | TCGA-COAD | Male | 74 | 26/174 | 0.053 | 64 |
| Q1367K | 1367 | Colon | TCGA-COAD | Female | 90 | 24/87 | 0.358 | 57 |
| A1377D | 1377 | Colon | TCGA-COAD | Male | 67 | 8/76 | 0.034 | 319 |
| G1137R | 1137 | Cervix uteri | TCGA-CESC | Female | 62 | 3/49 | 0.278 | 29 |
| R1181H | 1181 | Cervix uteri | CPTAC-3 | Female | 67 | 28/96 | 0.068 | 40 |
| T1117N | 1117 | Bronchus and lung | CPTAC-3 | Female | 51 | 47/293 | 0.121 | 73 |
| D1232Y | 1232 | Bronchus and lung | CPTAC-3 | Male | 54 | 44/123 | 0.153 | 383 |
| A1234T | 1234 | Bronchus and lung | TCGA-LUSC | Female | 69 | 11/112 | 0.149 | 77 |
| A1251D | 1251 | Bronchus and lung | TCGA-LUAD | Female | 53 | 12/30 | 0.29 | 45 |
| L1318M | 1318 | Bronchus and lung | TCGA-LUAD | Female | 52 | 26/64 | 0.098 | 116 |
| D1349E | 1349 | Bronchus and lung | HCMI-CMDC | Male | 40 | 33/75 | 0.207 | 230 |
| P1350T | 1350 | Bronchus and lung | TCGA-LUSC | Male | 78 | 12/42 | 0.283 | 119 |
| G1121D | 1121 | Breast | TCGA-BRCA | Female | 68 | 119/319 | 0.154 | 89 |
| R1209P | 1209 | Breast | TCGA-BRCA | Female | 90 | 60/400 | 0.227 | 33 |
| F1174L | 1174 | Bones, joints and articular cartilage of other and unspecified sites | TARGET-NBL | Female | 1 | 72/347 | 0.365 | 58 |
| M1138K | 1138 | Bladder | TCGA-BLCA | Male | 77 | 54/204 | 0.167 | 49 |
| I1171N | 1171 | Adrenal gland | TARGET-NBL | Female | 2 | 47/166 | 0.321 | 21 |
| F1174C | 1174 | Adrenal gland | TARGET-NBL | Male | 6 | 41/188 | 0.171 | 40 |
| F1174L | 1174 | Adrenal gland | TARGET-NBL | Male | 13 | 37/127 | 0.4 | 42 |
| F1174L | 1174 | Adrenal gland | TARGET-NBL | Female | 1 | 19/49 | 0.417 | 77 |
| F1174L | 1174 | Adrenal gland | TARGET-NBL | Male | 1 | 49/106 | 0.469 | 39 |
| L1240V | 1240 | Adrenal gland | TARGET-NBL | Male | 2 | 6/114 | 0.568 | 48 |
| R1275Q | 1275 | Adrenal gland | TARGET-NBL | Male | 3 | 24/67 | 0.252 | 69 |
| R1275Q | 1275 | Adrenal gland | TARGET-NBL | Male | 2 | 32/248 | 0.342 | 101 |
| R1275Q | 1275 | Adrenal gland | TARGET-NBL | Male | 8 | 11/321 | 0.506 | 80 |

In this table we can see ALK substitutions data available in TCGA database associated to different type of cancer, sex, age, MAF and depth sequencing value

**Supplementary table 3: ALK substitutions frequency associated with different type of cancer**

| **Type of cancer** | **%** | **N** |
| --- | --- | --- |
| Corpus uteri | 15.58% | 12 |
| Adrenal gland | 11.69% | 9 |
| Skin | 10.39% | 8 |
| Bronchus and lung | 9.09% | 7 |
| Colon | 9.09% | 7 |
| Other and ill-defined sites | 7.79% | 6 |
| Kidney | 6.49% | 5 |
| Uterus, NOS | 5.19% | 4 |
| Hematopoietic and reticuloendothelial systems | 3.90% | 3 |
| Breast | 2.60% | 2 |
| Cervix uteri | 2.60% | 2 |
| Unknown | 2.60% | 2 |
| Bladder | 1.30% | 1 |
| Bones, joints and articular cartilage of other and unspecified sites | 1.30% | 1 |
| Esophagus | 1.30% | 1 |
| Lymph nodes | 1.30% | 1 |
| Other and ill-defined sites in lip, oral cavity and pharynx | 1.30% | 1 |
| Other and unspecified parts of mouth | 1.30% | 1 |
| Ovary | 1.30% | 1 |
| Pancreas | 1.30% | 1 |
| Retroperitoneum and peritoneum | 1.30% | 1 |
| Stomach | 1.30% | 1 |

In this table we can see that ALK substitutions are mostly associated to corpus uteri, followed by adrenal gland, skin, bronchus, lung and colon cancer.

**Supplementary table 4: ALK substitutions display predicted SIFT and Polyphen-2 scores values and binding energy affinity with lorlatinib**

| **Nº** | **Amino acid substitution**  **ALK ^variant^** | **Amino acid position** | **SIFT Classification** | **SIFT Score** | **Polyphen impact** | **PolyPhen-2 score** | **Binding energy (Kcal/mol) ALK^variant^ - Lorlatinib** |
| --- | --- | --- | --- | --- | --- | --- | --- |
| 1 | ALK E1110K | 1110 | deleterious | 0 | possibly_damaging | 0.618 | -9.6 |
| 2 | ALK P1112Q | 1112 | deleterious | 0 | probably_damaging | 0.963 | -9.8 |
| 3 | ALK T1117N | 1117 | deleterious | 0.02 | possibly_damaging | 0.451 | -9.6 |
| 4 | ALK R1120W | 1120 | deleterious | 0 | probably_damaging | 0.996 | -9.6 |
| 5 | ALK G1121D | 1121 | deleterious | 0 | probably_damaging | 0.95 | -9.7 |
| 6 | ALK G1137R | 1137 | deleterious | 0.01 | probably_damaging | 0.995 | -9.8 |
| 7 | ALK E1161K | 1161 | deleterious | 0 | probably_damaging | 0.951 | -9.7 |
| 8 | ALK E1167A | 1167 | deleterious | 0 | probably_damaging | 0.986 | -9.7 |
| 9 | ALK I1171N | 1171 | deleterious | 0 | probably_damaging | 0.994 | -9.7 |
| 10 | ALK F1174L (G>T) | 1174 | deleterious | 0.03 | possibly_damaging | 0.641 | -9.7 |
| 11 | ALK F1174C | 1174 | deleterious | 0 | probably_damaging | 0.999 | -9.7 |
| 12 | ALK F1174I | 1174 | deleterious | 0 | probably_damaging | 0.988 | -9.7 |
| 13 | ALK F1174L (A>G) | 1174 | deleterious | 0.03 | possibly_damaging | 0.641 | -9.7 |
| 14 | ALK Q1177H | 1177 | deleterious | 0.01 | possibly_damaging | 0.885 | -9.7 |
| 15 | ALK R1181C | 1181 | deleterious | 0 | probably_damaging | 0.975 | -9.7 |
| 16 | ALK A1200V | 1200 | deleterious | 0 | possibly_damaging | 0.605 | -9.8 |
| 17 | ALK G1202E | 1202 | deleterious | 0.01 | probably_damaging | 0.996 | -10 |
| 18 | ALK G1202R | 1202 | deleterious | 0 | probably_damaging | 0.997 | -9.9 |
| 19 | ALK R1209P | 1209 | deleterious | 0 | probably_damaging | 0.995 | -9.7 |
| 20 | ALK R1209Q | 1209 | deleterious | 0.01 | probably_damaging | 0.976 | -9.7 |
| 21 | ALK R1212H | 1212 | deleterious | 0 | probably_damaging | 0.991 | -9.6 |
| 22 | ALK R1212C | 1212 | deleterious | 0 | probably_damaging | 0.994 | -9.6 |
| 23 | ALK P1213H | 1213 | deleterious | 0 | probably_damaging | 0.998 | -9.7 |
| 24 | ALK V1229A | 1229 | deleterious | 0 | probably_damaging | 0.954 | -9.7 |
| 25 | ALK D1232Y | 1232 | deleterious | 0 | probably_damaging | 1 | -9.7 |
| 26 | ALK A1234T | 1234 | deleterious | 0 | probably_damaging | 1 | -9.8 |
| 27 | ALK L1240V | 1240 | deleterious | 0 | probably_damaging | 0.993 | -9.7 |
| 28 | ALK E1242K | 1242 | deleterious | 0 | probably_damaging | 0.991 | -9.8 |
| 29 | ALK F1245I | 1245 | deleterious | 0 | probably_damaging | 1 | -9.8 |
| 30 | ALK A1251D | 1251 | deleterious | 0 | probably_damaging | 1 | -9.6 |
| 31 | ALK A1252V | 1252 | deleterious | 0 | probably_damaging | 0.997 | -10.8 |
| 32 | ALK R1253T | 1253 | deleterious | 0 | probably_damaging | 1 | -9.7 |
| 33 | ALK G1269E | 1269 | deleterious | 0 | probably_damaging | 1 | -10 |
| 34 | ALK R1275Q | 1275 | deleterious | 0 | probably_damaging | 1 | -9.7 |
| 35 | ALK R1279M | 1279 | deleterious | 0 | probably_damaging | 0.997 | -9.7 |
| 36 | ALK P1292S | 1292 | deleterious | 0 | probably_damaging | 1 | -9.7 |
| 37 | ALK G1304E | 1304 | deleterious | 0 | probably_damaging | 1 | -9.6 |
| 38 | ALK S1308Y | 1308 | deleterious | 0 | probably_damaging | 0.997 | -9.7 |
| 39 | ALK L1318M | 1318 | deleterious | 0.03 | probably_damaging | 1 | -9.7 |
| 40 | ALK S1324Y | 1324 | deleterious | 0 | probably_damaging | 1 | -9.7 |
| 41 | ALK Q1336H | 1336 | deleterious | 0 | probably_damaging | 0.99 | -9.7 |
| 42 | ALK V1338A | 1338 | deleterious | 0 | probably_damaging | 0.997 | -9.6 |
| 43 | ALK T1343S | 1343 | deleterious | 0 | possibly_damaging | 0.836 | -9.7 |
| 44 | ALK M1348I | 1348 | deleterious | 0 | probably_damaging | 0.976 | -9.6 |
| 45 | ALK D1349N | 1349 | deleterious | 0.03 | probably_damaging | 0.91 | -9.7 |
| 46 | ALK P1350T | 1350 | deleterious | 0 | probably_damaging | 0.91 | -9.7 |
| 47 | ALK K1352N | 1352 | deleterious | 0 | possibly_damaging | 0.807 | -9.8 |
| 48 | ALK P1355L | 1355 | deleterious | 0 | probably_damaging | 1 | -9.4 |
| 49 | ALK P1357H | 1357 | deleterious | 0 | probably_damaging | 1 | -9.7 |
| 50 | ALK Q1364P | 1364 | deleterious | 0 | probably_damaging | 0.984 | -9.7 |
| 51 | ALK P1370T | 1370 | deleterious | 0 | probably_damaging | 0.996 | -9.7 |
| 52 | ALK C1386G | 1386 | deleterious | 0 | probably_damaging | 0.999 | -9.7 |
| 53 | ALK P1398L | 1398 | deleterious | 0.01 | probably_damaging | 0.982 | -9.7 |

In this table we can see predicted algorithms as deleterious (SIFT) and damaging (Polyphen-2) for all 53 ALK substitutions, as binding energy scores docked with lorlatinib.

**Supplementary table 5: AlphaMissense and OncoKB Classification consensus exploring ALK substitutions reported in TCGA with SIFT and Polyphen-2 oncogenic predictors**

| **Item** | **Variant** | **Protein change** | **Position** | **Pathogenicity Score** | **pathogenicity classification** | **Changes in same position** | **OncoKB Classification** | **Relevant info from OncoKB** |
| --- | --- | --- | --- | --- | --- | --- | --- | --- |
| 1 | E1110K | p.Glu1110Lys | 1110 | 0.963 | likely_pathogenic | 19 | not found |  |
| 2 | P1112Q | p.Pro1112Gln | 1112 | - | not found the exact change | 13 | not found |  |
| 3 | T1117N | p.Thr1117Asn | 1117 | - | not found the exact change | 2 | not found |  |
| 4 | R1120W | p.Arg1120Trp | 1120 | 0.710 | likely_pathogenic | 15 | not found |  |
| 5 | G1121D | p.Gly1121Asp | 1121 | 0.980 | likely_pathogenic | 17 | not found |  |
| 6 | G1137R | p.Gly1137Arg | 1137 | - | not found the exact change | 1 | not found |  |
| 7 | E1161K | p.Glu1161Lys | 1161 | 0.971 | likely_pathogenic | 19 | not found |  |
| 8 | E1167A | p.Glu1167Ala | 1167 | 0.999 | likely_pathogenic | 19 | not found |  |
| 9 | I1171N | p.Ile1171Asn | 1171 | 0.989 | likely_pathogenic | 16 | Likely Oncogenic | Resistant to crizotinib, ceritinib, and alectinib; sensitive to brigatinib and lorlatinib in vitro and clinically. (45) |
| 10 | F1174L (G>T) | p.Phe1174Leu | 1174 | 1.000 | likely_pathogenic | 19 | Oncogenic | resistance to crizotinib, ceritinib, and alectinib. However, it demonstrates sensitivity to lorlatinib and brigatinib in preclinical and clinical settings (45) |
| 11 | F1174C | p.Phe1174Cys | 1174 | 0.996 | likely_pathogenic | 19 | Likely Oncogenic | mutation shows resistance to crizotinib and ceritinib, but is sensitive to alectinib, brigatinib, and lorlatinib, as demonstrated in preclinical studies and a patient case (45). |
| 12 | F1174I | p.Phe1174Ile | 1174 | 0.997 | likely_pathogenic | 19 | Likely Oncogenic | In vitro studies show it is sensitive to crizotinib, ceritinib, alectinib, and lorlatinib (45). |
| 13 | F1174L (A>G) | p.Phe1174Leu | 1174 | 1.000 | likely_pathogenic | 19 | Oncogenic | resistance to crizotinib, ceritinib, and alectinib. However, it demonstrates sensitivity to lorlatinib and brigatinib in preclinical and clinical settings (45). |
| 14 | Q1177H | p.Gln1177His | 1177 | - | not found the exact change | 1 | not found |  |
| 15 | R1181C | p.Arg1181Cys | 1181 | - | not found the exact change | 6 | not found |  |
| 16 | A1200V | p.Ala1200Val | 1200 | - | not found the exact change | 2 | Likely neutral |  |
| 17 | G1202E | p.Gly1202Glu | 1202 | 0.995 | likely_pathogenic | 19 | not found |  |
| 18 | G1202R | p.Gly1202Arg | 1202 | 0.986 | likely_pathogenic | 19 | Resistance | resistance to crizotinib, ceritinib, alectinib, and brigatinib, but shows sensitivity to lorlatinib (45). |
| 19 | R1209P | p.Arg1209Pro | 1209 | 0.999 | likely_pathogenic | 19 | not found |  |
| 20 | R1209Q | p.Arg1209Gln | 1209 | 0.914 | likely_pathogenic | 19 | not found |  |
| 21 | R1212H | p.Arg1212His | 1212 | 0.967 | likely_pathogenic | 19 | not found |  |
| 22 | R1212C | p.Arg1212Cys | 1212 | 0.978 | likely_pathogenic | 19 | not found |  |
| 23 | P1213H | p.Pro1213His | 1213 | 0.911 | likely_pathogenic | 19 | not found |  |
| 24 | V1229A | p.Val1229Ala | 1229 | - | not found the exact change | 13 | not found |  |
| 25 | D1232Y | p.Asp1232Tyr | 1232 | 0.987 | likely_pathogenic | 19 | not found |  |
| 26 | A1234T | p.Ala1234Thr | 1234 | 0.800 | likely_pathogenic | 17 | Likely Neutral |  |
| 27 | L1240V | p.Leu1240Val | 1240 | 0.937 | likely_pathogenic | 19 | not found |  |
| 28 | E1242K | p.Glu1242Lys | 1242 | 0.633 | likely_pathogenic | 13 | Likely Neutral |  |
| 29 | F1245I | p.Phe1245Ile | 1245 | 0.999 | likely_pathogenic | 19 | not found |  |
| 30 | A1251D | p.Ala1251Asp | 1251 | 1.000 | likely_pathogenic | 19 | not found |  |
| 31 | A1252V | p.Ala1252Val | 1252 | 0.994 | likely_pathogenic | 19 | not found |  |
| 32 | R1253T | p.Arg1253Thr | 1253 | 1.000 | likely_pathogenic | 19 | not found |  |
| 33 | G1269E | p.Gly1269Glu | 1269 | 1.000 | likely_pathogenic | 19 | not found |  |
| 34 | R1275Q | p.Arg1275Gln | 1275 | 0.991 | likely_pathogenic | 19 | Oncogenic | found in familial neuroblastoma, demonstrates sensitivity to ALK inhibitors crizotinib and lorlatinib in both in vitro and in vivo studies (45). |
| 35 | R1279M | p.Arg1279Met | 1279 | 0.999 | likely_pathogenic | 19 | not found |  |
| 36 | P1292S | p.Pro1292Ser | 1292 | 1.000 | likely_pathogenic | 19 | not found |  |
| 37 | G1304E | p.Gly1304Glu | 1304 | 1.000 | likely_pathogenic | 19 | not found |  |
| 38 | S1308Y | p.Ser1308Tyr | 1308 | 0.901 | likely_pathogenic | 15 | not found |  |
| 39 | L1318M | p.Leu1318Met | 1318 | 0.854 | likely_pathogenic | 19 | not found |  |
| 40 | S1324Y | p.Ser1324Tyr | 1324 | 0.997 | likely_pathogenic | 19 | not found |  |
| 41 | Q1336H | p.Gln1336His | 1336 | 0.940 | likely_pathogenic | 18 | not found |  |
| 42 | V1338A | p.Val1338Ala | 1338 | 0.995 | likely_pathogenic | 19 | not found |  |
| 43 | T1343S | p.Thr1343Ser | 1343 | 0.584 | likely_pathogenic | 16 | not found |  |
| 44 | M1348I | p.Met1348Ile | 1348 | 0.997 | likely_pathogenic | 18 | not found |  |
| 45 | D1349N | p.Asp1349Asn | 1349 | 0.869 | likely_pathogenic | 19 | not found |  |
| 46 | P1350T | p.Pro1350Thr | 1350 | 0.912 | likely_pathogenic | 18 | not found |  |
| 47 | K1352N | p.Lys1352Asn | 1352 | 0.933 | likely_pathogenic | 17 | not found |  |
| 48 | P1355L | p.Pro1355Leu | 1355 | 0.982 | likely_pathogenic | 19 | not found |  |
| 49 | P1357H | p.Pro1357His | 1357 | 0.971 | likely_pathogenic | 19 | not found |  |
| 50 | Q1364P | p.Gln1364Pro | 1364 | 0.988 | likely_pathogenic | 18 | not found |  |
| 51 | P1370T | p.Pro1370Thr | 1370 | 0.897 | likely_pathogenic | 19 | not found |  |
| 52 | C1386G | p.Cys1386Gly | 1386 | 0.913 | likely_pathogenic | 19 | not found |  |
| 53 | P1398L | p.Pro1398Leu | 1398 | 0.890 | likely_pathogenic | 18 | not found |  |
|  |  |  |  |  |  |  |  |  |
| **References: > 0.56: Likely_pathogenic and < 0.56: Likely_benign** | | | | | | | | |
