# Supplementary figures and images for: An in silico evaluation of lorlatinib as a potential therapy for novel amino acid substitutions in the tyrosine kinase domain of the ALK protein associated with cancer
Source: Front Pharmacol. 2025 Jun 18;16:1605314. doi: 10.3389/fphar.2025.1605314 (PMC12213726; doi:10.3389/fphar.2025.1605314)

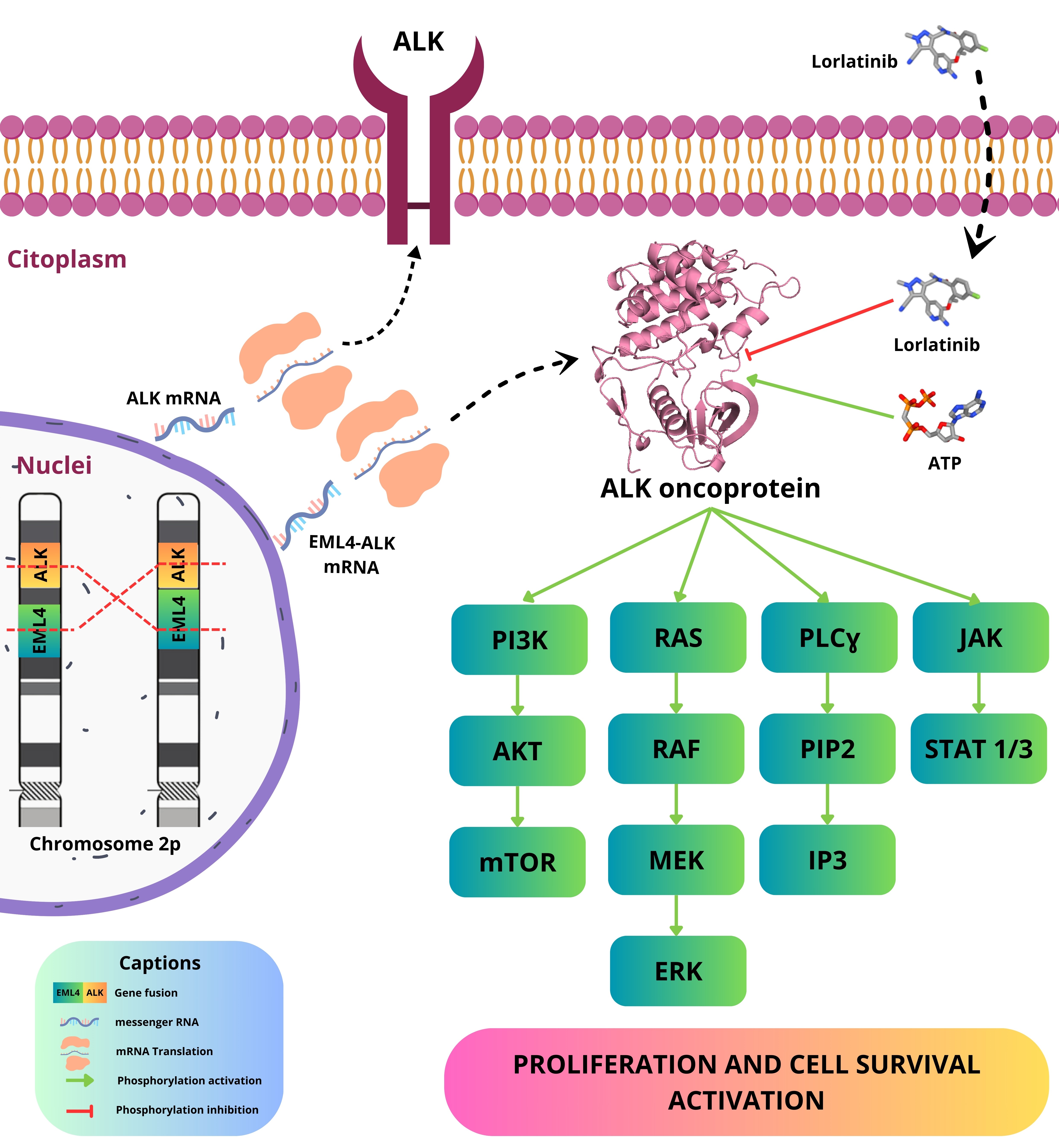

Supplement: Supplementary file 2 [file Image1.jpeg]

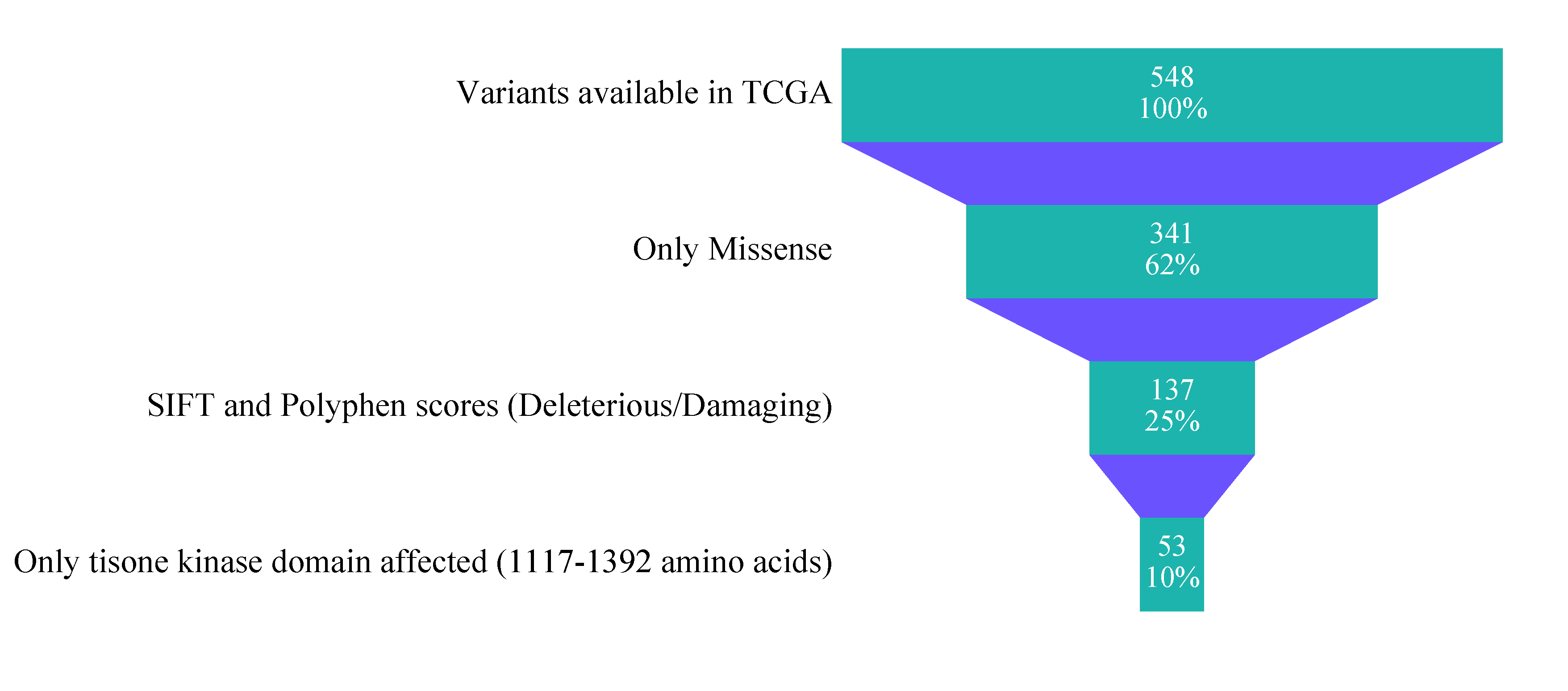

Supplement: Supplementary file 3 [file Image2.png]
